# Supplementary figures and images for: Planarian EGF repeat-containing genes megf6 and hemicentin are required to restrict the stem cell compartment
Source: PLoS Genet. 2020 Feb 20;16(2):e1008613. doi: 10.1371/journal.pgen.1008613 (PMC7059952; doi:10.1371/journal.pgen.1008613)

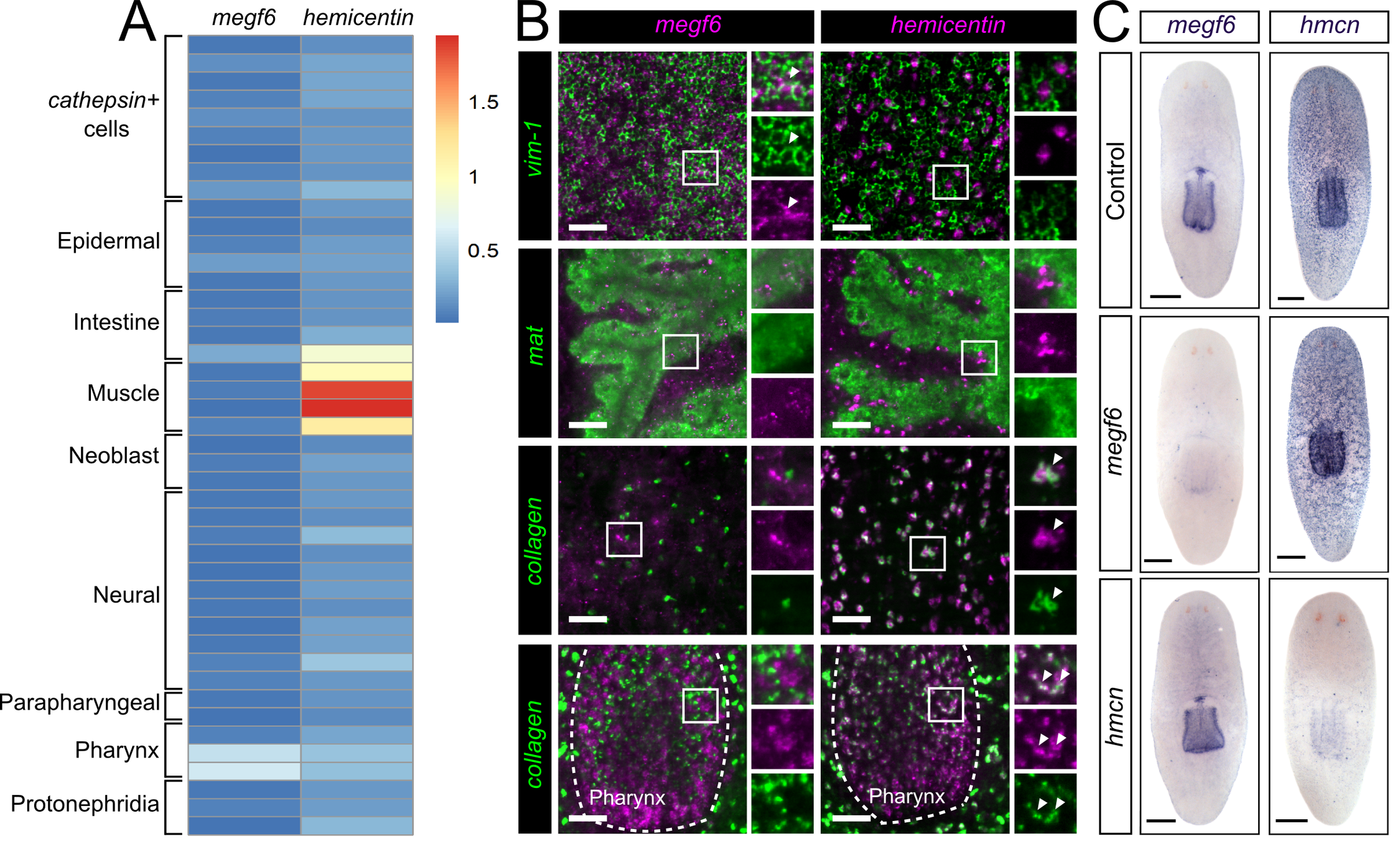

Supplement: S1 Fig — A) Heatmap showing the expression of megf6 and hemicentin in different tissues from single-cell RNA sequencing (data from Fincher et al. 2018 and downloaded from https://compgen.bio.ub.edu/PlanNET/planexp). B) Double fluorescent in situ hybridization for megf6 or hemicentin with markers of the epidermis (vim-1), intestine (mat), and muscle (collagen) in wild-type planarians. White boxes denote the magnified region shown on the right, and white arrows show cells with expression of both genes. C) Colorimetric whole mount in situ hybridization (WISH) stains for megf6 and hemicentin in control, megf6, or hemicentin RNAi animals. Scale bars: 50 μm in B; 250 μm in C. (TIF) [file pgen.1008613.s001.tif]

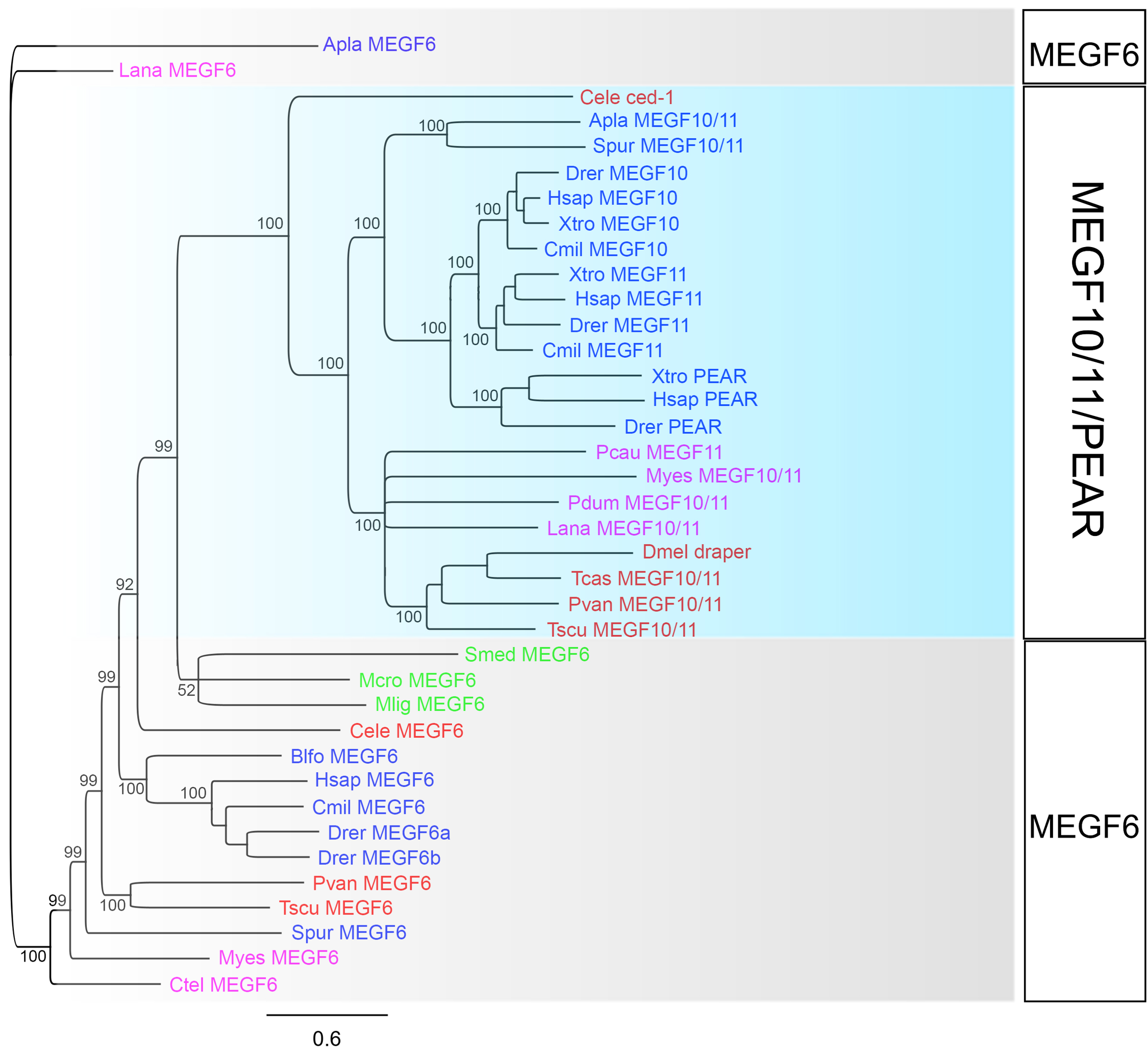

Supplement: S2 Fig — Names in blue are Deuterostomes, names in red are Ecdysozoans, names in magenta are Lophotrochozoans, and the flatworms are in green. All important node support values are listed as % support next to the relevant node. All MEGF10/11/PEAR/Draper/ced-1 proteins cluster together with 100% support (blue shaded box). A full list of protein sequences and accession numbers can be found in Supplemental File 1. Species names: Lana = Lingula anatine[brachiopod]; Pcau = Priapulus caudatus[priapulid]; (flatworms) Smed = Schmidtea mediterranea; Mcro = Maritigrella crozieri[polyclad]; Mlig = Macrostomum lignano[basal flatworm]; (Ecdysozoa) Cele = Caenorabditis elegans; Dmel = Drosophila melanogaster; Tcas = Tribolium castaneum[flour beetle]; Pvan = Penaeus vannamei[shrimp]; Tscu = Centruroides sculpturatus[scorpion]; (Lophotrochozoa) Pdum = Platynereis dumerilii[polychaete]; Myes = Mizuhopecten yessoensis[mollusk]; Ctel = Capitella teleta[polychaete]; (Dueterostomes) Apla = Acanthaster planci[starfish]; Spur = Strongylocentrotus purpuratus[sea urchin]; Cmil = Callorhinchus milii[shark]; Blfo = Branchiostoma floridae[lancelet]; Xtro = Xenopus tropicalis; Hsap = Homo sapiens; Drer = Danio rerio. (TIF) [file pgen.1008613.s002.tif]

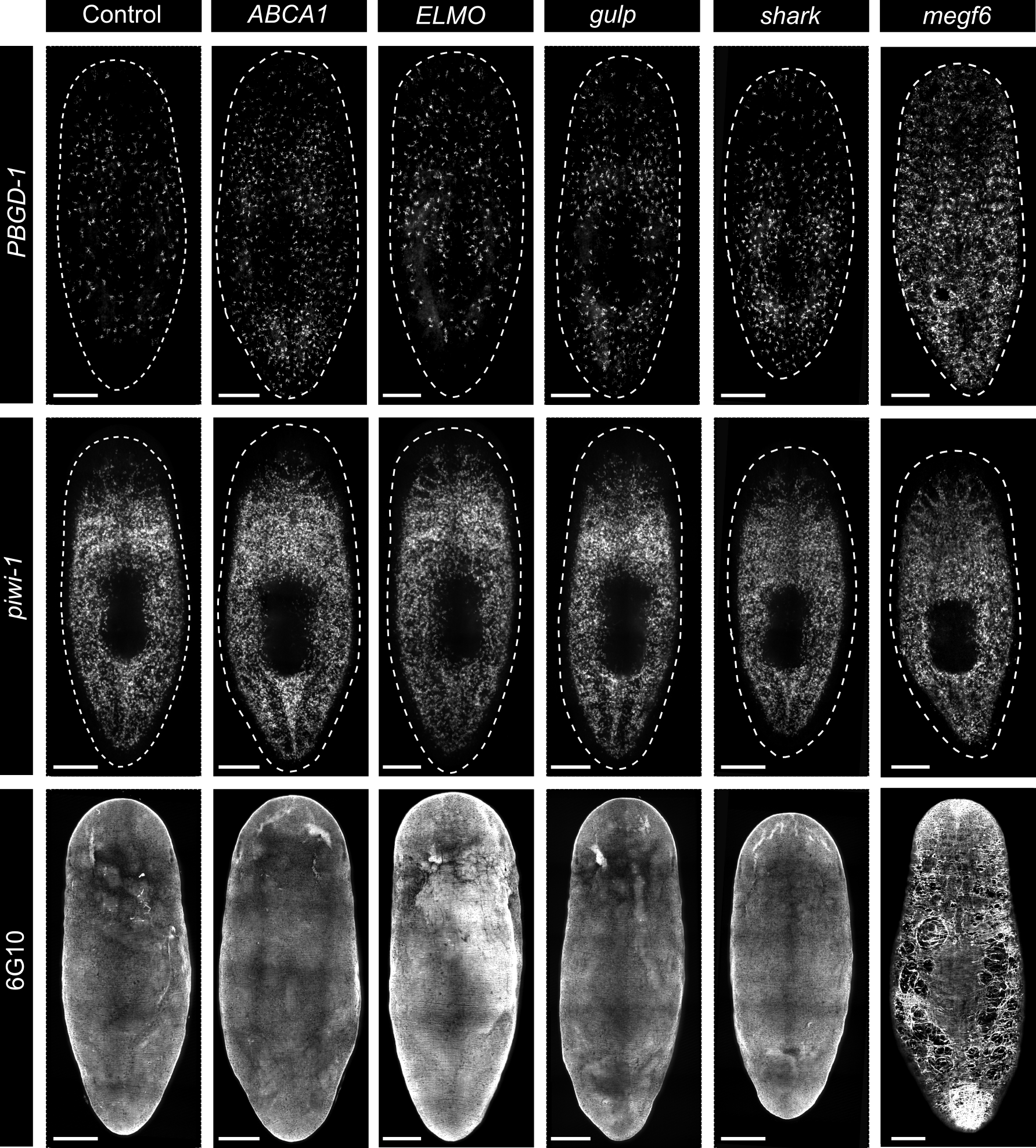

Supplement: S3 Fig — Single confocal planes showing whole mount fluorescent in situ hybridization (FISH) for PBGD-1, FISH for piwi-1, and immunostaining for muscle fibers with 6G10 antibody (n ≥ 8). Draper pathway knockdown animals are compared with controls (left) as well as megf6 knockdown planarians (right). Scale bars are 250 μm. (TIF) [file pgen.1008613.s003.tif]

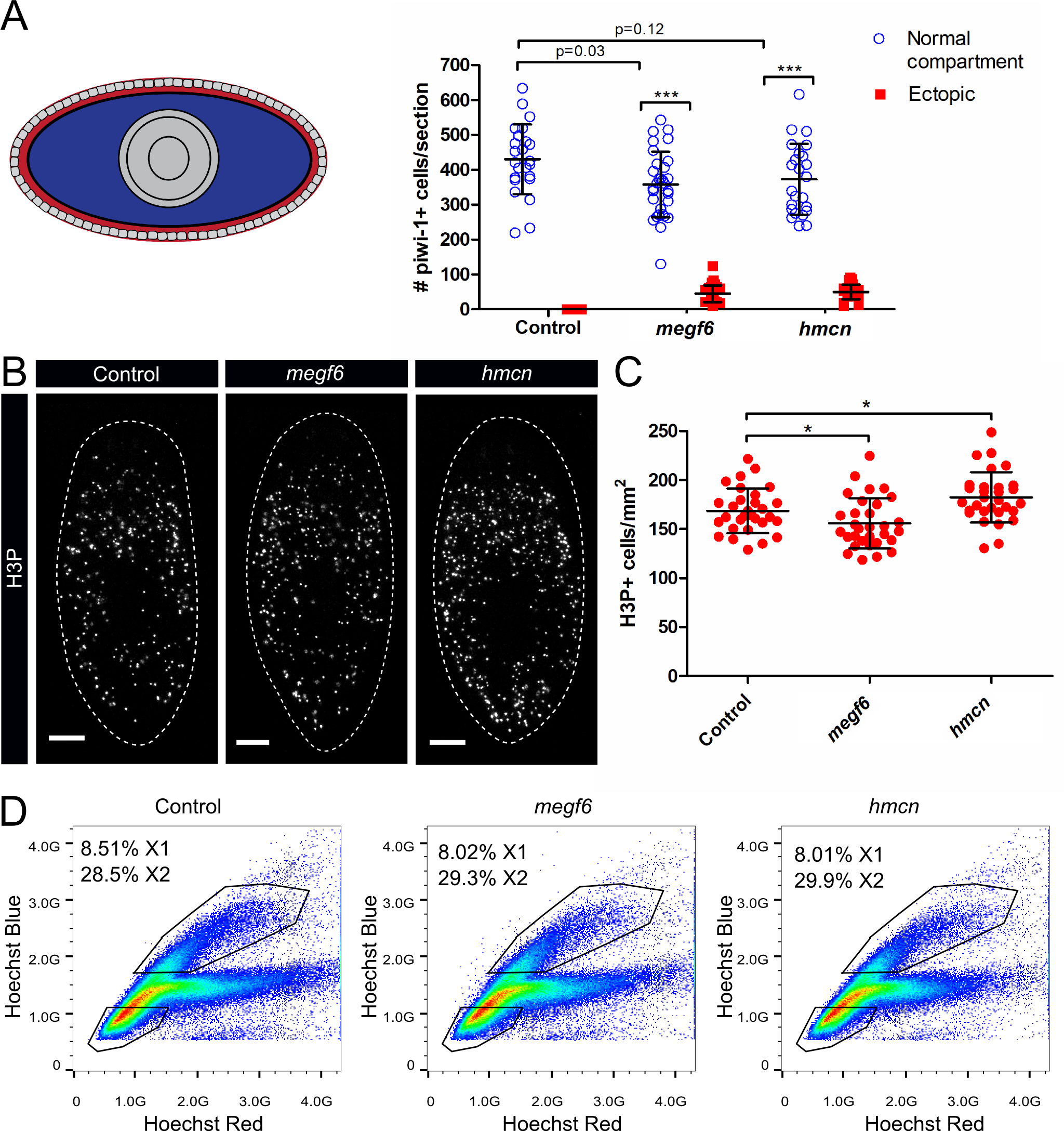

Supplement: S4 Fig — A) Quantification of piwi-1 cells in transverse cross-sections shown in Fig 3A (n ≥ 24). Cross-sections were taken from the same axial regions of control and knockdown animals. Diagram shows the regions quantified (left). B) Whole mount staining for phosphorylated-histone H3 on serine 10 (H3P) in control, megf6, or hemicentin knockdown worms (n ≥ 30). C) Quantification of the number of mitoses, measured from the whole animal as shown in A). D) FACS plots of Hoechst-stained cells from control, megf6, or hemicentin knockdown worms. The X1 gate contains actively cycling neoblasts with >2n DNA content. Proportions of cells in the X1 and X2 gates are shown on each plot. Scale bars are 250 μm. Error bars are standard deviation. *p < 0.05, ***p<0.001 (Welch’s t-test). (TIF) [file pgen.1008613.s004.tif]

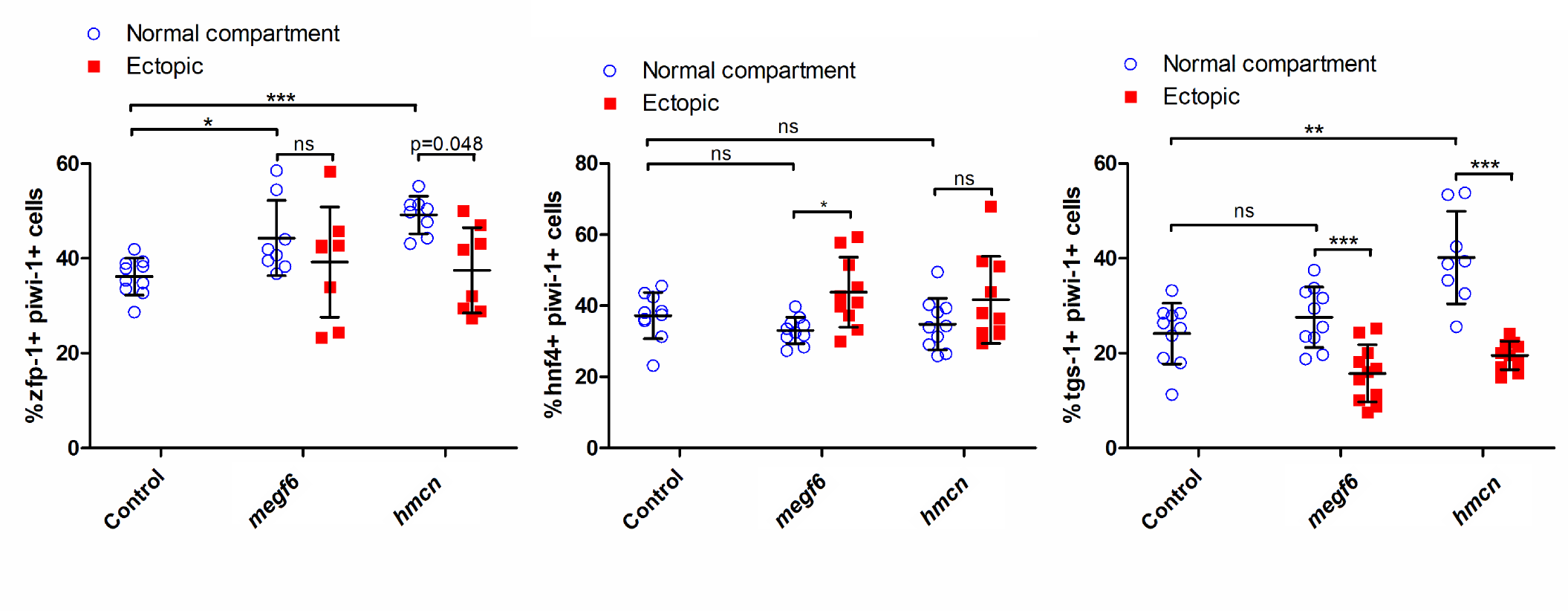

Supplement: S5 Fig — Percentage of piwi-1+ cells co-expressing marker genes of the zeta (zfp-1), gamma (hnf4), or putative pluripotent (tgs-1) neoblast subclasses in control and knockdown planarians (n ≥ 8). Quantifications are of dFISH images as shown in Fig 3D, with ‘normal’ and ‘ectopic’ compartments taken from different confocal planes equivalent to the regions shown in Fig 3B. Error bars are standard deviation. *p < 0.05, **p < 0.01, ***p<0.001 (Welch’s t-test). (TIF) [file pgen.1008613.s005.tif]

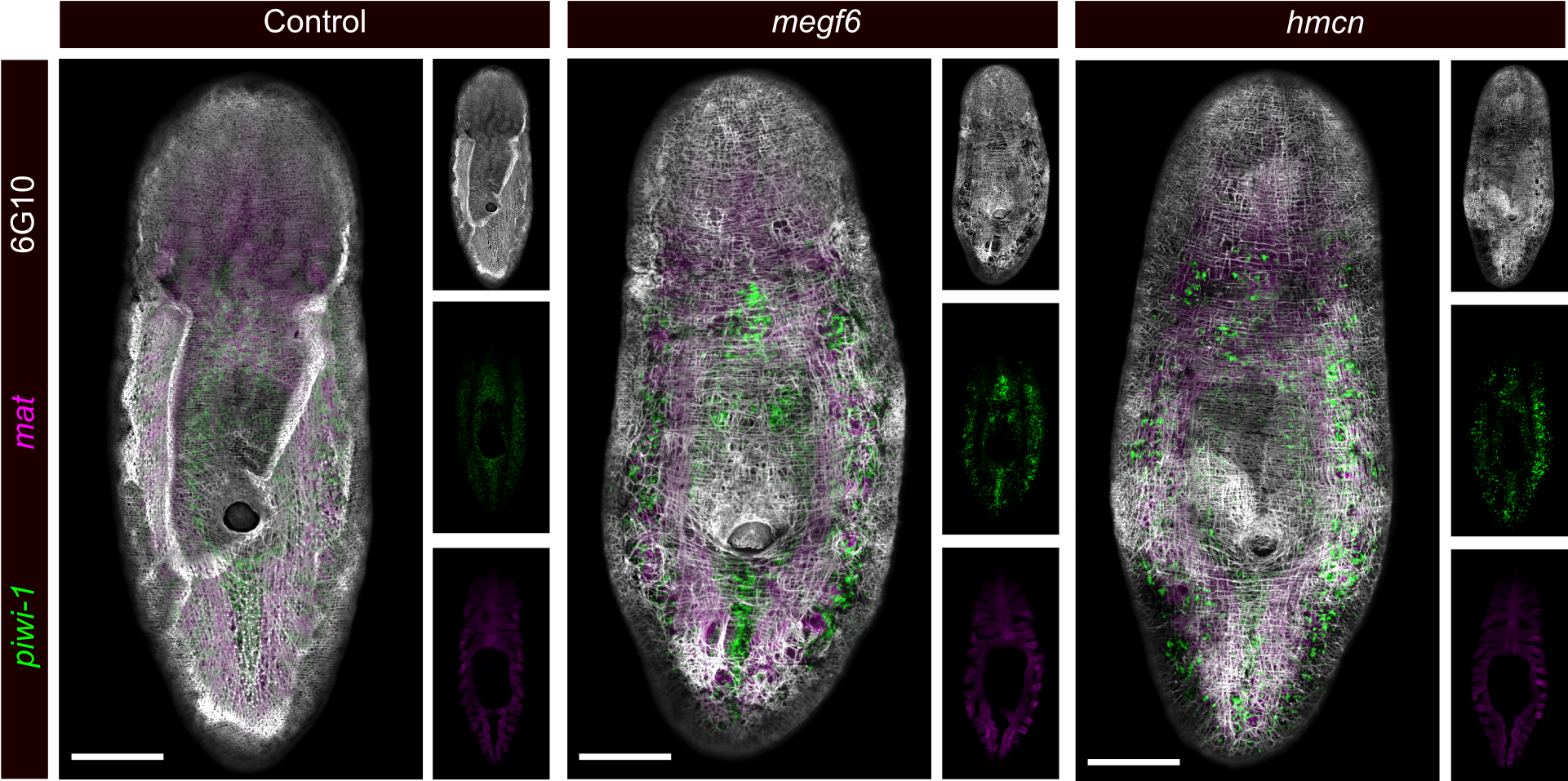

Supplement: S6 Fig — Single confocal planes showing a ventral view of control, megf6 or hemicentin knockdown planarians (n ≥ 10). The animals are stained with double FISH for piwi-1 and mat and immunostained with 6G10 to mark muscle fibers. Scale bars are 250 μm. (TIF) [file pgen.1008613.s006.tif]

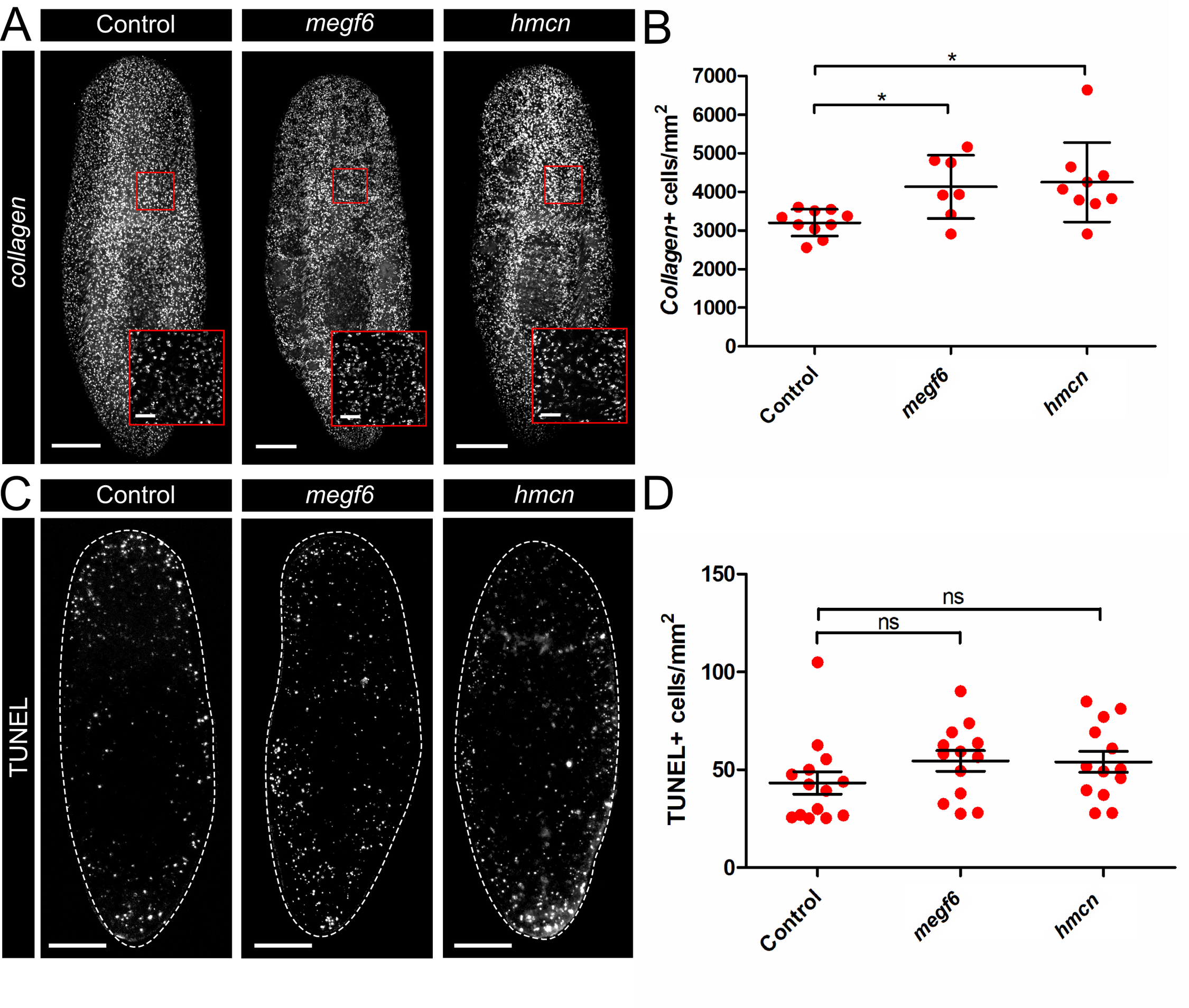

Supplement: S7 Fig — A) WISH of collagen-1, marking the cell bodies of body wall muscle. Red boxes mark the magnified regions shown in the insets. B) Quantification of collagen+ cells/mm2 in control, megf6, or hemicentin knockdown animals (n ≥ 7). Cell densities were measured from 20x tiles as shown in the insets of A. C) Whole worm images of TUNEL staining in control and knockdown planarians. D) Quantification of TUNEL+ cells from whole worm images shown in C (n ≥ 13). Scale bars are 250 μm, 100 μm for insets. Error bars are standard deviation. *p<0.05, ns = not significant (Welch’s t-test). (TIF) [file pgen.1008613.s007.tif]

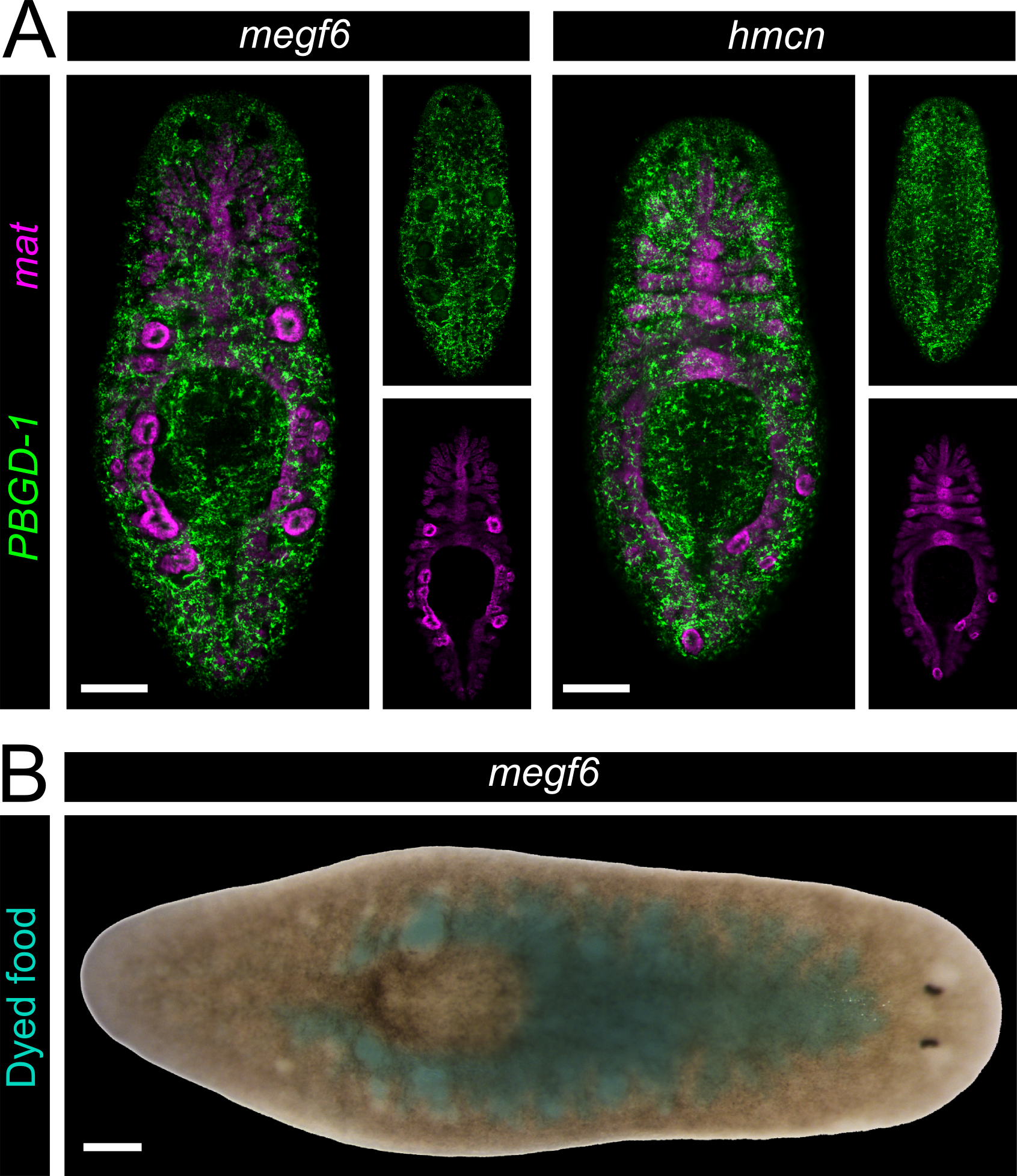

Supplement: S8 Fig — A) Double FISH for PBGD-1 and mat in megf6 or hemicentin knockdown planarians (n ≥ 6). Single confocal planes at the dorsal side of the animal are shown. B) Live image of a megf6 knockdown planarian after feeding with dyed liver (n = 1). The worm is shown dorsal side up, anterior to the right. Scale bars are 250 μm. (TIF) [file pgen.1008613.s008.tif]

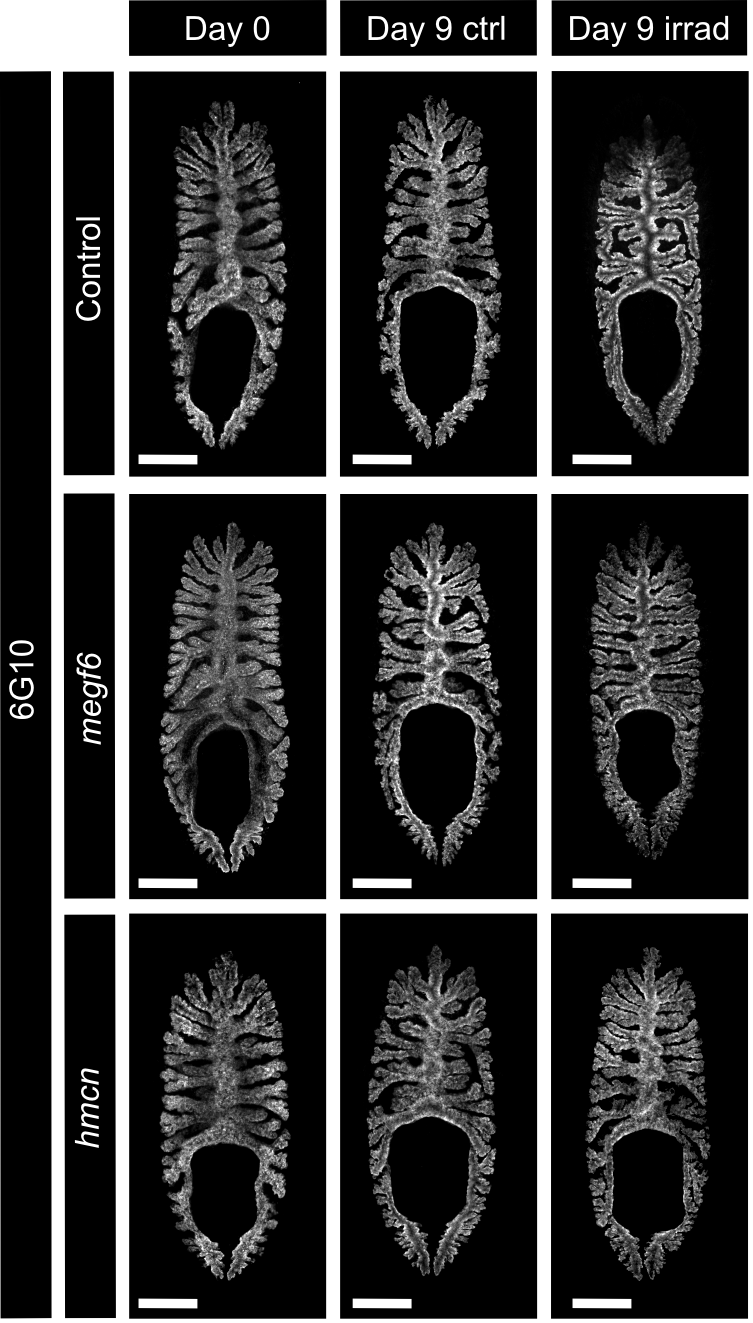

Supplement: S9 Fig — Single confocal planes showing the intestine, marked by in situ hybridization for mat, in animals from the experimental timepoints shown in Fig 5D (n ≥ 19). Scale bars are 250 μm. (TIF) [file pgen.1008613.s009.tif]

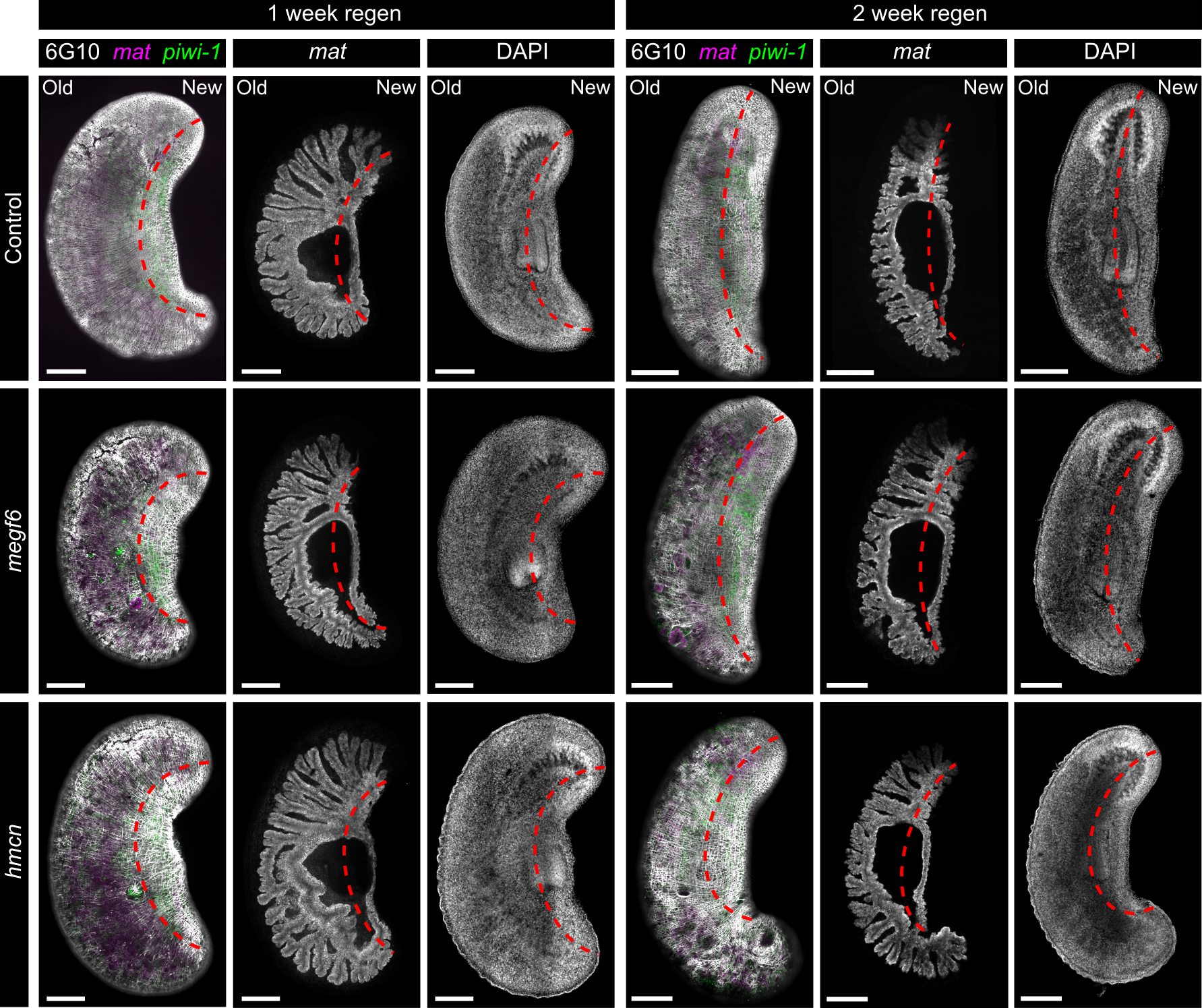

Supplement: S10 Fig — Optical sections of regenerating fragments 1 or 2 weeks following sagittal amputation (n ≥ 3). Structures shown are muscle fibers (6G10 immunostaining), neoblasts (FISH for piwi-1), intestine (FISH for mat), brain and pharynx (DAPI). Amputation planes are denoted with dotted red lines. Scale bars are 250 μm. (TIF) [file pgen.1008613.s010.tif]
