# Supplementary material for: Planarian EGF repeat-containing genes megf6 and hemicentin are required to restrict the stem cell compartment
Source: PLoS Genet. 2020 Feb 20;16(2):e1008613. doi: 10.1371/journal.pgen.1008613 (PMC7059952; doi:10.1371/journal.pgen.1008613)
Supplement: Attachment — Submitted filename: MEGF6_rebuttal.docx [file pgen.1008613.s013.docx]

**We would like to thank all reviewers for their positive comments and support of our work, as well as for their very helpful suggestions. The comments we received have helped us to greatly improve the manuscript, and we believe we have been able to address the major criticisms raised by the reviewers. We look forward to a review of our revised manuscript. Here is a bulleted list of new data added.**

**1) Phylogenetic analyses of the MEGF family has indeed produced very interesting results with MEGF6 loss in the insects, MEGF10/11 loss in the flatworms, yet both MEGF6 and MEGF10/11 present in pre-bilaterians (Fig S2). We have more accurately changed the name of the gene here to *Smed-megf6* and these data represent some of the only functional data on a MEGF6 gene.**

**2) We have performed a more detailed examination of the expression of *megf6* and *hemicentin*, including a heat map showing tissue-specific gene expression and double *in situ* hybridization experiments (Fig S1). These data show that *megf6* is expressed in the pharynx and epidermis, while *hemicentin* is expressed in *collagen+* muscle cells, including muscle cells located in the pharynx.**

**3) BrdU pulse-chase experiments have shown that ectopic *piwi-1* cells undergo DNA replication during a 24-hour period (Fig 3C).**

**4) We examined expression of *tgs-1* in ectopic stem cells and quantification of the number of ectopic neoblasts expressing *tgs-1*, *hnf4,* and *zfp-1* compared to neoblasts within the normal compartment (Fig 3D; Fig S5). This experiment shows that fewer ectopic neoblasts express *tgs-1* compared to their counterparts within the niche, suggesting that that a higher proportion may be differentiating.**

**5) We performed quantification of total *piwi-1* cell number in the cross-sections shown in Fig 3 and flow cytometry analysis of control and knockdown animals (Fig S4). These data show that the number of ectopic neoblasts is very small compared to the number of neoblasts in the normal niche, and that the total number of neoblasts in S/G2/M phase is not overtly affected by *megf6* or *hemicentin* knockdown.**

**6) Concanavalin A staining combined with muscle antibody and DAPI staining shows how the epidermis is affected in knockdown animals (Fig 4D). These stains indicate that ectopic cells associated with large muscle lesions invade the epidermis of the worm and disrupt the normal pattern of epidermal junctions.**

**7) We performed TUNEL staining to mark apoptotic cells in control and knockdown planarians (Fig S7). These data show that the number of apoptotic cells does not significantly increase with *megf6* or *hemicentin* knockdown.**

**8) Cross-sections of knockdown planarians at the ‘early’ timepoint (3 days after the 5^th^ RNAi feed) show that gut morphology is normal at the timepoint when ectopic stem cells begin to appear (Fig 5B).**

**9) We have added new transmission electron microscopy (TEM) experiments at 5fd3 to determine the attachment of muscle fibers to the basement membrane (Fig 5C). These images show that the basement membrane is thinner at this timepoint in knockdown planarians compared to controls, and muscle fibers are located farther than normal from the basement membrane.**

**10) We have included live images and *in situ* analysis of regenerating sagittal fragments 1 and 2 weeks post amputation (wpa). Live images show that the epidermis is wrinkled only in old tissue and that eyespots regenerate in both control and knockdown planarians by 2 wpa (Fig 6). Whole-mount images of muscle antibody staining combined with *in situ* hybridization for *mat* and *piwi-1* show that muscle lesions, ectopic gut, and ectopic stem cells appear only in old tissue up to 2wpa, and that muscle and gut both regenerate normally (Fig S10). Whole-mount DAPI stains show regeneration of the pharynx and brain (Fig S10).**

**Point by point specific revisions are below.**

Reviewer #1: This manuscript focuses on the role of extracellular matrix in planarians, and investigates the role of ECM in controlling tissue organization in planarians. Planarian muscle is the source of many, if not all, ECM proteins (as shown in Cote et al, 2019). Here, the authors investigate the role of EGF-containing proteins draper and hemicentin. Hemicentin has been shown previously to constrain distribution of stem cells and their progeny. Knockdown of draper results in a virtually identical phenotype to hemicentin RNAi, where stem cells and intestinal branches are no longer constrained by the ECM/muscle periphery, and instead leak out beyond the ECM. It is very interesting that the expression of draper is so different from hemicentin, yet their knockdown phenotypes are so similar. The authors document this phenotype with beautiful, clear imaging of several disrupted cell types. The stem cells, which are normally restricted to the interior of the body adjacent to intestines, now appear on the outside of the body and continue to proliferate there. The authors suggest that stem cells initiate rupture through the muscle layer, because stem cells emerge prior to the intestinal branches, and the lesioned area is slightly less in irradiated than in control animals. Overall, this is a very intriguing manuscript with very clear and thorough documentation of the phenotypes. However, some of the conclusions are slightly overstated and would benefit from clarification as suggested below.

Major comments:

It remains unclear what is the primary driver of the phenotype. The radiation experiment suggests that stem cells play some role, but the subtlety of the phenotype suggests that other factors are involved too. It seems like the primary difference between the new and old tissue is that muscle fibers no longer attach to the ECM. One way to characterize this further would be to determine when the intestinal branches emerge out of the newly regenerated tissue, and examine the muscle/ECM proximity by EM prior to onset of that phenotype. If muscle and ECM are still adjacent to each other, then the authors can more conclusively state that the stem cells drive the phenotype.

**We agree that the stem cells are likely not the only factor involved in the development of the phenotypes. Cote et al. have previously observed that epidermal wrinkling and ectopic cells are observed following lethal irradiation in *hemicentin* RNAi, and in line with this, we did observe both epidermal wrinkling and some disorganization of muscle fibers (as shown in Fig 5A and 5D) in irradiated knockdown animals. Therefore, stem cells are not the driver of these phenotypes. We suspect that muscle disorganization caused by a different mechanism, independent of stem cells, may contribute to the formation of these lesions as well, though our irradiation experiment demonstrates that stem cells at least accelerate this process. We have made a note of the results observed by Cote et al. in line 310. Importantly, we did not observe muscle lesions in regions of the worm with low numbers of stem cells (above the pharynx and in the anterior), further suggesting that stem cells drive muscle lesions.**

**To assess the timing of muscle lesion detachment from the ECM, we examined the basement membrane by EM prior to emergence of ectopic gut branches in planarians at the ‘early’ stage phenotype (rather than in regenerating planarians as you suggested, in order to limit the time frame of the experiment). We found that the basement membrane appears thinner in knockdown animals than in controls, and muscle fibers have already begun to detach from the ECM (new data in Fig 5C). Few ectopic stem cells have appeared at this timepoint, so we cannot conclude that ectopic stem cells drive detachment of muscle fibers from the ECM.**

Stem cells are often located adjacent to the intestine, so as the intestine ruptures through the muscle wall, the stem cells may come along passively, giving the impression that they are leading an invasive event. Is there a way to rule out this possibility?

**To rule out this possibility, we have included cross-sections of knockdown worms at the ‘early’ stage phenotype to examine gut morphology (new data in Fig 5B). We observed no dorsal gut outgrowths at this time, even directly below emerging ectopic stem cells. Additionally, we have included images of gut morphology from the animals in our irradiation experiment (new data in Fig S9), and we observed no ectopic gut branches in any of these worms. Finally, in late-stage RNAi animals, ectopic stem cells and muscle lesions are observed in locations where no ectopic gut branches are observed (Fig 4). Based on these observations, we conclude that ectopic stem cells are not carried passively by the intestine, though we cannot rule out that signals from the gut may be altered in our RNAi conditions.**

The conclusion that draper and hemicentin knockdown animals display hyperproliferation is poorly supported. The methods for quantification should be better explained to justify how the “ectopic niche” is distinguished from a “normal niche” – for example, is it restricted to the outer epidermis? Without this information, it is not clear, at least to this reviewer, what the criteria for hyperproliferation were. The authors should clarify this issue, particularly because in the control image (3A), there is proliferation occurring just below the epidermis.

**The distinction between ‘normal’ and ‘ectopic’ compartments was made using DAPI staining, with the gap in nuclei (indicating the region occupied by muscle fibers) serving as the boundary. Thus, the H3P+ cell in Fig 3A, located below this gap in the DAPI staining, was counted as part of the ‘normal’ compartment. We have added diagrams in Fig 3B to clarify this.**

In other organisms, Draper functions in apoptotic cell engulfment. However, it seems to have a different role in planarians – no transmembrane domain, and a phenotype distinct from that of other known engulfment proteins. In this sense, it almost seems disingenuous to refer to this gene as a Draper homolog, and instead the authors may consider referring to it as MEGF6.

**We have adopted this new terminology as you suggested. We would like to thank you for the suggestion, as further investigation into the phylogeny of this gene has shown that the gene family has had an unappreciated and interesting evolutionary history (new analysis in Fig. S2).**

The radiation experiment should include intestinal morphology as a control.

**These stains have been added in Fig S9.**

The authors should mention if/when the animals die, and how this relates to the number of RNAi feeds administered. Also, population sizes and penetrance need to be indicated.

**This information has been added to the text in line 124, and population sizes have been added to figure legends.**

Reviewer #2: The manuscript by Lindsay-Mosher et al. entitled “Planarian EGF repeat-containing genes draper and hemicentin are required to restrict the stem cell compartment” examines the role of the ECM in planarians stem cell regulation. Knockdown of the aforementioned EGF-containing genes results phenotypes wherein uninjured planarians develop epidermal wrinkling and the formation of unpigmented regions/spots on the dorsal side. Analysis of the cellular processes underlying the observed phenotypes indicate that the ECM components studied in this paper play a role in restricting the localization of stem cells in the parenchyma. Absence of draper and hemicentin leads to an ectopic accumulation of stem cells as well as the ectopic differentiation of intestinal branches. Overall, this is a very nice story founded on beautiful and compelling data, which also supports findings reported in a recent screen by Cote et al. However, the paper is descriptive in its current form, and lacks a clear hypothesis and strong conclusions. In my opinion, additional experiments will be necessary to substantiate some of the conclusions and provide some mechanistic insight into the role of the ECM in neoblast regulation. Furthermore, some of the terminology co-opted in the paper seemed inadequate. My concerns and suggestions for improving the paper are described below.

Major concerns:

The abstract and introduction hint at a goal of uncovering mechanisms restricting cell division and the localization of the planarian neoblasts. But the manuscript focuses on describing the phenotypes rather than analyzing the source of the ectopic neoblasts or the signaling mechanisms leading to the expansion and clustering of the stem cell population. I think the authors are correct in stating that the stem cell niche is not well defined in planarians. Knocking these two ECM components (in contrast to other factors, like beta1-integrin), convincingly affects the stem cell population. It would be much more insightful to explore which stem cell subtypes are affected by the knockdown and whether there are truly more neoblasts (if there is hyperproliferation) or ectopic/mispositioned stem cells. Thus, the authors present interesting ideas regarding understanding how an [undefined] niche restricts localization of the stem cell population, which likely indirectly impacts their regulation (i.e., cell division rates, differentiation signals) but do not explore this problem in depth. The types of experiments that could be performed include in situs for neoblast subtypes, flow cytometry measurements, and TUNEL (which was conspicuously missing from the post-RNAi analysis where lesions are described).

**We agree that a more in-depth examination of differentiation signals inside vs outside the normal neoblast compartment would be an important addition to this story. To address this, we quantified the proportions of *piwi-1+* neoblast expressing the lineage markers *zfp-1*, *hnf-4*, and the pluripotency marker *tgs-1* in both the normal neoblast compartment and ectopic locations (i.e. basal vs apical to the muscle layer). We found that all three markers were found in the ectopic neoblast clusters, but their proportions were altered based on location (new data in Fig 3D, Fig S5). These results suggest that neoblasts outside of the normal compartment do receive altered differentiation signals, though we are unable to identify the source of these cues. Further speculation on these results has been added to the Discussion section (line 422).**

**To address the question of cell cycle regulation, we performed a 24 hour BrdU pulse-chase and found that a large proportion of ectopic *piwi-1* cells were BrdU+. Unfortunately, we were unable to quantify this experiment due to high regional variability in the BrdU staining, but we were able to conclude that the vast majority of ectopic neoblasts are capable of DNA replication (new data in Fig 3C). To assess whole-animal proliferation, we performed flow cytometry with Hoechst staining and quantified the X1 gate (>2n DNA content). We found that the proportion of cells in the X1 gate was not overtly altered in our RNAi conditions, in line with our previous data from whole-worm H3P staining which showed only very slight changes in whole-animal proliferation (new data in Fig S4). From these data, we conclude that on the level of the whole animal, *megf6* and *hemicentin* RNAi do not cause hyperproliferation. This was not surprising, as the number of proliferating, ectopic stem cells was very small compared to the number of stem cells within the normal compartment, and therefore, altered cell cycle dynamics of these neoblasts are unlikely to significantly affect whole-worm proliferation. We also performed TUNEL staining, and found no significant increase in cell death in knockdown worms (new data in Fig S7).**

Specific comments/suggestions:

1) The description of draper homology and its expression could be improved. The authors vacillate between ascribing draper expression to pharynx cells and suggesting it is likely in other cell types. The t-SNE plot reveals that draper is highly expressed in other cell types. What are they? The lack of clarity about draper localization becomes problematic for the conclusions and the model. As the authors describe in the Discussion, it doesn’t make much sense that pharynx expression would cause the observed phenotype. Furthermore, I didn’t see a solid phylogenetic analysis for draper, which combined with the evidence described in the paper (e.g., previous literature, lack of similar expression to other draper genes and function, and the domain structure) indicate to me that the gene analyzed in this paper is not really a draper ortholog.

**We have clarified the cell-type expression of *megf6* and *hemicentin* in Fig S1. These results show that *megf6* is expressed in the epidermis as well as the pharynx, while *hemicentin* is expressed in muscle cells. We have performed a phylogentic analysis as detailed above and in the new Fig S2 with some interesting conclusions.**

From what I gathered in the Discussion, the authors could consider naming this gene Smed-megf6, which according to the description of the structure appears to be a better fit for the planarian homolog. I think the Pearson lab is adept at analyzing gene evolutionary relationships. Such analysis is a glaring omission from this paper.

**We agree with this suggestion and have changed the gene name accordingly. We would like to thank you for holding us to it!**

2) The authors describe the formation of white spots/unpigmented regions on the dorsal surface as lesions. Can the authors visualize epidermal openings? To address this distinction between their conclusions and Cote et al., the authors could stain RNAi knockdown planarians with fluorescent-Concanavalin A to visualize the epidermal surface of the animals in those regions. From the data shown here and in the Cote manuscript, I would refrain from calling the spots lesions.

**We performed Concanavalin A staining as suggested, and found that epidermal junctions are disrupted in regions containing ectopic gut branches (new data in Fig 4D). However, DAPI staining shows that cells are present in these areas, and these ‘lesions’ do not cause worm death (unlike the lesions that form when epidermal differentiation is disrupted). Therefore, to avoid confusion, we now refer to these regions as “unpigmented spots”, though we do still refer to “muscle lesions”.**

3) The paragraph discussing epidermal progression after RNAi: I think the authors should be cautious with the interpretation that the “progression” of epidermal differentiation is disrupted. From the data presented I would conclude localization of the cells is disrupted, unless experiments are performed to assess progression (e.g., BrdU pulse-chase labeling experiments).

**It was not our intention to claim that epidermal lineage progression is altered in the RNAi conditions, and we agree that the language we used was confusing. We have changed the section to specify that localization of these cells within the parenchyma is disrupted, as you concluded.**

4) Section on ectopic neoblast hyper-proliferation and Figure 3: this is a key figure in this paper and where I think the authors would consider performing more in-depth analyses. The observations that knocking down “draper” and hemicentin leads to ectopic neoblast localization and that mis-localized neoblasts have proliferative potential are not debatable and are consistent with observations from the Reddien lab. However, the intriguing possibility that ectopic neoblasts divide at a higher rate is not as convincing (Figure 3B and Figure S6). One of the most exciting observations in the manuscript is the formation of piwi-1+ clusters in RNAi knockdown conditions. This suggests that there are more neoblasts in the regions analyzed and I suspect one is more likely to detect dividing cells within those clusters. Unfortunately, it is difficult to assess how this analysis was performed. The authors could show images of the regions analyzed and should normalized the cell counts by counting the number of piwi-1+ cells within the region of interest and the area or volume (unless the regions of interest quantified were the same between control and experimental worms). A related question that I have is whether there are more stem cells in ECM RNAi worms, which could be assessed by performing flow cytometry analysis; if so, this would provide strong support for a hyper-proliferation phenotype. Additional experiments that would augment the impact of this part of the story would be to include labeling for stem cell subtypes following RNAi. For example, does the distribution of tspan-1+ (or another marker like tgs-1) stem cells change and could it explain how the formation of ectopic neoblast “colonies” form? Do they also associate with ectopic gut branches? Finally, I am a little skeptical about the observations presented in Figure S6. If one ignores the statistical tests, there is not a robust difference in cell division rates. I was also wondering why the authors chose to perform a Welch’s t-test for this data, which I believe is most appropriate when samples have unequal samples sizes and/or variances. If they were to perform a standard Student’s t-test, are the differences still significant? In any case, my interpretation in the whole animal analysis is that although significant (as shown), there is no strong support for global changes in proliferation rates if one were to compare these phenotypes to others that clearly lead to altered cell division rates and expansion of the neoblast population, a parameter that could be analyzed by FACS.

**We agree with your interpretation that *megf6* and *hemicentin* RNAi do not overtly affect global proliferation rates. To more clearly show how the analysis of proliferation in Fig 3B was performed, we have added diagrams to illustrate the locations of the quantified cells. The counts shown in Fig 3B were taken from the cross-sections shown in Fig 3A, at the same region in control and knockdown planarians, and H3P+ cell counts are all normalized to the number of *piwi-1+* cells in the corresponding region. We argue that these data show that ectopic stem cells in *megf6* and *hemicentin* RNAi are hyperproliferating; however, proliferation within the normal stem cell compartment occurs at a similar or slightly lower rate compared to controls. We have added the control data to Fig 3B to make this comparison. As you have pointed out, the whole-animal H3P staining shows only very slight changes in the rate of proliferation between control and knockdown animals (Fig S4). We believe this is because the total number of stem cells, and therefore mitoses, within the ectopic compartment is very small compared to the number of stem cells within the normal compartment of knockdown planarians (new data in Fig S4). Therefore, the total number of proliferating stem cells within the worm does not change dramatically. We performed flow cytometry as you suggested, and found that the number of cells within the X1 gate (in S/G2/M) is very similar between control and knockdown worms, supporting this hypothesis (new data in Fig S4).**

**We chose to perform a Welch’s *t*-test for all quantifications in this work because this method is more stringent than a Student’s *t*-test in the case of samples with unequal variance, and functions approximately the same as a Student’s *t*-test when variance is equal. To our understanding, there is no disadvantage to broadly using a Welch’s *t*-test in place of the Student’s *t*-test. In the case of our whole-animal proliferation values, a Student’s *t*-test produces the same result.**

**We have examined the distribution of *tgs-1* in knockdown animals as you suggested and found that *tgs-1* is expressed in ectopic stem cell clusters (new data in Fig 3D). Interestingly, we found that significantly fewer ectopic stem cells express *tgs-1* compared to their counterparts in the normal compartment, perhaps due to increased distance from the normal gut (new data in Fig S5). We also found slightly increased *hnf4* expression in the ectopic stem cells, potentially explaining the formation of the ectopic gut branches (new data in Fig S5). Further speculation on this has been added to the Discussion section (line 422).**

5) What is the normal niche? I don’t think this is well-defined in planarians, other than localization of neoblasts near the intestinal branches. I would consider omitting the word niche when describing the phenotype.

**We agree with this point, and have changed our wording to “normal compartment” instead of “normal niche”.**

6) Although there is evidence for ectopic cell localization (as in tumors) to cause lesions, I am not convinced about the use of this word throughout the paper (as mentioned in Comment #2). What is the evidence the cells are “lesioning” the muscle? Are they simply causing muscle to be disorganized or to become remodeled as a result of ectopic differentiation of other organs, like the intestine? Moreover, Figure S8 shows an increase in the number of collagen+ cells. The authors mention that this could be also due to an increase in muscle cell death. Although I am not aware if the muscle antibody is compatible with the TUNEL assay, the authors could test if there is a global or regional increase in cell death.

**We agree that the ectopic stem cells could be remodeling the muscle fibers rather than causing muscle cell death. Here, we use the word ‘lesion’ to describe an abnormal gap in the muscle fibers, not one necessarily caused by cell death. We have no evidence to suggest that any ectopic organs aside from the gut are differentiating at the sites of muscle lesions, and ectopic gut branches are found at only a few lesion sites (Fig 4). We performed TUNEL staining in knockdown animals and found no significant increase in apoptosis, suggesting that muscle cell death is not a major factor in the development of this phenotype (new data in Fig S7).**

Minor points:

1) The author summary contains a few typos.

**Fixed.**

2) The Methods section is unacceptably superficial. There is no explanation of how cell counts were performed for Figure 3B or for the triple-FISH experiments shown in Figure 2.

**We have added more detail in the Methods section (line 531) and included more information on the quantifications in Fig 3B.**

Reviewer #3: In this manuscript entitled “Planarian EGF repeat-containing genes draper and hemicentin are required to restrict the stem cell compartment,” Lindsay-Mosher et al. expand our understanding of the diverse functions of ECM components in regulating stem cells. Using the planarian Schmidtea mediterranea as a model, the authors have investigated two genes, draper and hemicentin, which putatively encode ECM proteins required for basement membrane integrity, organization of the sub-epidermal muscle layer, and restriction of localization/growth of stem cells, stem cell progeny, intestine, and eyes in uninjured animals. Both genes encode EGF repeat-containing proteins. Consistent with their phenotypes, hemicentin is expressed in pharynx and subepidermal muscle, while draper is expressed in pharynx and epidermal cells, apparently throughout the animal. hemicentin’s phenotype was previously described in a genome-wide analysis of the planarian matrisome (Cote et al., Nat. Comm., 2019); Lindsay-Mosher and colleagues’ studies largely replicate and validate this previous study, but with additional analysis and insights (e.g., hyperproliferation of mis-localized neoblasts, ectopic growth of gut and eyes were reported in the current manuscript). The studies of draper are novel. Intriguingly, its knockdown phenocopies hemicentin RNAi (including epidermal ruffling, muscle disorganization, and body wall lesions), suggesting draper has a role in maintaining ECM and tissue boundaries, rather than in phagocytosis, its better characterized role in other model organisms. The authors conduct very detailed analyses of the draper and hemicentin phenotypes, and suggest their data indicate that (a) draper and hemicentin restrict migration/growth of multiple tissues, (b) that piwi-1-positive neoblasts “cause” ectopic outgrowths, (c) that draper, hemicentin, and normal ECM are dispensable for at least seven days of regeneration, and (d) that the “normal” niche in planarians inhibits proliferation.

The manuscript is clearly written, experiments are conducted rigorously, and figures are clear and organized. The study will be of interest to the planarian research community, since only a few studies have identified genes that restrict the stem cell compartment, and this model is ideally suited to address this understudied problem. Furthermore, the study should also attract broader interest by those interested in ECM biology and how ECM influences stem cell dynamics and tissue regeneration. However, the authors are encouraged to consider several concerns, both major and minor, that would significantly improve the manuscript if addressed. These issues include the depth of analysis of whether these genes are dispensable for regeneration, the ultimate cause of body wall lesions, interpretation of draper’s roles with respect to phagocytosis vs. functioning as an ECM component, and interpretation of whether the “normal” niche restricts proliferation.

Major concerns:

(1) Are draper and hemicentin really dispensable for regeneration? The authors’ conclusion is based on quite superficial characterization of the RNAi phenotypes in new tissue, especially given that the ECM is not normal in new tissue (Fig. 6D-E). For example, although Fig. 6A shows regeneration of anterior and posterior muscle, it is not clear if epidermis, brain, eyes, or intestine regenerate properly. Similarly, although the experiment in Fig. 6B (sagittal amputation) is elegant, it is not clear if muscle, epidermis, brain, eyes, intestine, or pharynx regenerate properly. The authors only show that ectopic pigment cells arise at 2 weeks in the new tissue (even though close inspection suggests there might be a small number of externally located pigment cells at 1 week). At a minimum, the authors should show images of epidermis (is there evidence of wrinkling?) and eyes from animals in Fig. 6A, and whole mount images of muscle labeling in new vs. old tissue in Fig. 6B, as well as images of epidermis and eyes. It would also be straightforward to assess CNS and pharynx regeneration in sagittally amputated animals with DAPI labeling. While possibly beyond the scope of the manuscript, obviously in situ hybridization would be the best way to assess regeneration of other organs, and/or whether ectopic stem cells and/or progeny emerge earlier.

**We have included more detail on the regeneration of the sagittal fragments in our experiments. Live images of the regenerating fragments show epidermal wrinkling only in the new tissue, and show that new eyespots regenerate in the knockdown planarians (new data in Fig 6). We have also added whole-animal images of muscle labelling combined with *piwi-1* and *mat*, and have taken your suggestion to show pharynx and CNS regeneration with DAPI (new data in Fig S10).**

(2) Do piwi-1-positive neoblasts really cause body wall lesions? The authors attempt to determine the cause of body wall lesions by conducting a detailed time course of the emergence of phenotypes in draper and hemicentin RNAi animals, and by ablating stem cells using irradiation. However, the authors do not seem to consider the possibility that stem cell progeny in the epidermal lineage (e.g., prog-2-positive, agat-3-positive), and not stem cells themselves, are responsible. This seems likely given that prog-2 and agat-3-positive cells (and not piwi-1-positive neoblasts) normally must migrate through sub-epidermal muscles and basement membrane, making them likely candidates to initiate body wall lesions through secretion of MMPs or other processes. In Fig. 2B, many more prog-2-positive and agat-3-positive cells are observed in the epidermis and subepidermis in draper and hemicentin knockdowns, supporting this idea. The authors should test this possibility experimentally, or soften their interpretation and consider this possibility in the results and discussion.

**We agree with this interpretation and have added this possibility to the discussion (line 433). We think it is likely that progeny of the epidermal lineage may contribute to muscle disorganization and basement membrane degradation, as these cells must pass the muscle layer to become epidermal cells (discussed in line 449). However, *prog-2+* and *agat-3+* cells do not form clusters in the absence of ectopic *piwi-1*+ cells (Fig 2). We argue that stem cells must ultimately be driving the muscle lesion phenotype, whether directly or indirectly through the production of progeny. We did attempt to test the role of progeny cells in the development of the ECM phenotype using *mex3* knockdown to block differentiation, but unfortunately, the animals died before observable phenotypes developed in any group.**

(3) Does dysregulation of phagocytosis contribute to the draper phenotype? The authors suggest that draper’s main function is to regulate ECM, since knockdown of other genes with putative roles in phagocytosis does not have the same phenotype, and since draper does not encode a transmembrane domain. However, this is not a formal demonstration that draper is or is not required for phagocytosis. Isn’t it possible that failure to phagocytose old (senescent?) or ectopically located cells may contribute to ECM degradation and/or muscle disorganization? Have the authors tested whether old or dying cells accumulate in draper RNAi animals? The authors might want to consider this idea more extensively in their Discussion, or preferably, test it directly.

**The phylogenetic analysis we have included shows that the gene we initially thought was *draper* is *megf6* (new Fig S2), which has no published role in phagocytosis. We cannot rule out that *megf6* may play a role in phagocytosis, but we did perform TUNEL staining on our knockdown animals and found no significant increase in apoptosis as a result of *megf6* or *hemicentin* RNAi (new data in Fig S7). We conclude that apoptotic cells are unlikely to contribute to the development of the phenotypes.**

(4) Does the “normal” niche restrict proliferation? The authors report that proliferation (based solely on H3P-S10 labeling) is elevated in the “ectopic” niche (which is only vaguely defined by the authors, Fig. 3B, see below in minor concerns), while proliferation is unaffected in the “normal” niche (again, only vaguely defined). Based on this, the authors suggest (in the abstract, intro, and discussion) that the “normal” niche and/or the basement membrane itself may suppress stem cell proliferation. The authors seem to consider this the only interpretation of their results. Given that no molecule that suppresses or promotes proliferation in the “ectopic” niche has been identified, and that proliferation is unaffected in the “normal” niche despite the fact that some piwi-1-positive cells are located immediately basal to the muscle layer, the authors’ interpretation might be perceived as highly speculative to some readers. While the observations are intriguing, it might be prudent to consider additional explanations in discussion. For example, isn’t it possible that disorganized/ectopic muscle cells secrete pro-proliferative cues inappropriately? Or that ectopic gut branches (or visceral muscle) provide growth-promoting nutrients or other cues (considered superficially on line 385)? Or, if draper does regulate phagocytosis, that elevated populations of dying cells might promote proliferation?

**The alternative interpretations outlined here are all good suggestions. We have added some experimental evidence to address these possibilities: we show that ectopic stem cells appear in the absence of ectopic gut (new data in Fig 5B), and that the number of apoptotic cells is not significantly increased in knockdown animals (new data in Fig S7). We also show that ectopic *piwi-1*+ cells incorporate BrdU during a 24-hour chase (new data in Fig 3C), although we were unable to quantify this experiment due to very high variability in the BrdU staining. However, we agree that we cannot conclusively state that suppressive signals within the niche prevent hyperproliferation based on our data, and have added more discussion of these caveats (line 420, line 427).**

Minor concerns:

(1) Lines 206-207 & Fig 3A-B. The authors should more clearly (and quantitatively, if possible) define “normal” vs. “ectopic” niche regions on the sections in which cells were quantified, preferably with a diagram. Were neoblasts basal to the basement membrane included as ectopic, or not? Experimental precision here is very important to the authors’ conclusions regarding a possible suppressive role for the niche.

**We have added diagrams to clarify this issue (Fig 3B). Only neoblasts apical to the body wall muscle layer (assessed by the gap in the DAPI staining) were included as ectopic.**

(2) Greater detail is required in Fig. 5C for how “% muscle area lesioned” was quantified, since this is a quantitative experiment. Also, values for the control animal should be included.

**Control animals were used as a reference for this experiment, such that only gaps in the muscle larger than any seen in controls were counted. Therefore, all values for control animals were 0% area lesioned. We have included these details in the methods section (line 527) and figure caption.**

(3) More detail in methods is required for determining homology of planarian draper to its Drosophila and human homologs. Was this determination made via BLAST? If so, which regions are responsible for homology? What settings were used in SMART to identify different domains? Is it possible to provide a molecular phylogenetic tree?

**We have provided a phylogenetic analysis as suggested. We would like to thank you for pointing us in this direction!**

(4) Why are there increased muscle cells? Is this due to increased differentiation, or reduced cell death? This might warrant brief discussion. Also, are increased muscle cells observed ventrally?

**We have added a short discussion of this issue (line 275).**

(5) Fig. S7. An image of the ventral muscles in a control animal should be provided.

**This image has been added (new data in Fig S6).**

(6) Lines 154-157, lines 176-186, and/or in Fig. 5B. Did the authors observe wrinkled epidermis in irradiated animals, as in Cotes et al. - reference [26]? Although a minor issue, this might warrant brief discussion, since it would seem that epidermal detachment from the BM occurs independently of ectopic migration of neoblasts and their progeny.

**We performed lethal irradiation at a timepoint later than Cotes et al., when epidermal wrinkling had already begun to develop. We have added a brief discussion of the previously published data in line 310.**

(7) Lines 245-246. The authors mention that the gut branches were the “cause” of the white lesions observed in the animals. Would it be better to say that the lesions “are” gut branches visible immediately below the epidermis, since the authors suggest that neoblast “cause” the lesions (notwithstanding Major concern #2 above)?

**We have changed the wording accordingly.**

(8) Fig. 1 and 6. Would the authors consider including a cross-section schematic of the defects to accompany the TEM images for readers unfamiliar with planarian anatomy?

**We have included a diagram in Fig 1.**

(9) What is the ultimate fate of draper and hemicentin RNAi animals? Do they die/lyse?

**Surprisingly they do not! We have added this information to the results section (line 124).**
